# Lifestyle can exert a significant impact on the development of metabolic comorbidities in early-stage colorectal cancer patients

**DOI:** 10.3389/fnut.2025.1551526

**Published:** 2025-07-04

**Authors:** Yu Xin, Chunxia Liu, Jianfang Cui, Yanan Wang, Honglei Wu

**Affiliations:** ^1^Department of Gastroenterology, The Second Hospital, Cheeloo College of Medicine, Shandong University, Jinan, Shandong, China; ^2^Department of Gastroenterology, People’s Hospital of Xiajin County, Dezhou, Shandong, China; ^3^Department of Gastroenterology, Beijing Shijitan Hospital, Beijing, China

**Keywords:** early-stage colorectal cancer, metabolic syndrome, type 2 diabetes mellitus, metabolic dysfunction-associated fatty liver disease, lifestyle

## Abstract

**Background:**

Metabolic dysregulation has been identified as contributing to colorectal cancer (CRC) development. However, there is a lack of data regarding the association between lifestyle factors and metabolic diseases in CRC patients.

**Methods:**

We conducted a multi-center cross-sectional study including 437 early-stage CRC patients and 437 control participants between April 2023 and March 2024. The dietary inflammatory index (DII) was calculated based on dietary data, which was collected using a food frequency questionnaire. A healthy lifestyle was defined as adherence to an anti-inflammatory diet (DII score < 0) combined with active physical activity.

**Results:**

Among early-stage CRC patients, overweight and obesity were associated with an anti-inflammatory diet (OR = 0.585, 95% CI = 0.346–0.988, *p* = 0.045; OR = 0.463, 95% CI = 0.221–0.966, *p* = 0.040). Metabolic syndrome (MS) was associated with overweight or obesity (OR = 2.203, 95% CI = 1.283–3.782, *p* = 0.004) and age (OR = 1.052, 95% CI = 1.030–1.073, *p* < 0.001). Type 2 diabetes mellitus (DM2) or prediabetes was associated with overweight or obesity (OR = 1.788, 95% CI = 1.079–2.960, *p* = 0.024) and age (OR = 1.053, 95% CI = 1.032–1.073, *p* < 0.001). Metabolic dysfunction-associated fatty liver disease (MAFLD) was associated with overweight or obesity (OR = 1.807, 95% CI = 1.122–2.910, *p* = 0.015), age (OR = 1.039, 95% CI = 1.020–1.058, *p* < 0.001), and an unhealthy lifestyle (OR = 4.314, 95% CI = 1.549–12.014, *p* = 0.005). Moreover, both an active lifestyle and a healthy lifestyle were significantly associated with a lower likelihood of being diagnosed with overweight or obesity, MS, DM2 or prediabetes, and MAFLD (*p* < 0.05). Stratified analysis revealed that late-onset CRC patients adhering to an active lifestyle and a healthy lifestyle showed risk reductions for these metabolic comorbidities (*p* < 0.05).

**Conclusion:**

Adherence to healthy lifestyles, particularly in individuals aged ≥50 years, may alleviate metabolic dysregulation in early-stage CRC patients.

## 1 Introduction

Colorectal cancer (CRC) is the third most common cancer in the world (1), and the fourth leading cause of cancer-related deaths in China (2). Over the past decade, the morbidity and mortality of CRC is rising in our country (3). This trend is partially attributed to the rising prevalence of obesity and associated metabolic comorbidities, such as metabolic syndrome (MS), type 2 diabetes mellitus (DM2), and metabolic dysfunction-associated fatty liver disease (MAFLD). Obesity is recognized as a critical risk factor for CRC, primarily due to its role in promoting chronic low-grade systemic inflammation. Specifically, adipose tissue, especially visceral fat, secretes pro-inflammatory cytokines like tumor necrosis factor-alpha (TNF-*α*) and interleukin-6 (IL-6), which contribute to insulin resistance and may facilitate tumorigenesis in colorectal tissues (4–6). In addition to systemic inflammation, gut microbiota dysbiosis has emerged as a pivotal factor in CRC development. An imbalance in the gut microbial community can disrupt the mucosal barrier, enhance intestinal permeability, and activate immune responses, leading to a pro-inflammatory environment conducive to carcinogenesis. Notably, specific pathogenic bacteria, such as enterotoxigenic *Bacteroides fragilis* and certain *Escherichia coli* strains, have been implicated in CRC pathogenesis (7).

Lifestyle factors, including diet and physical activity, significantly influence both systemic inflammation and gut microbiota composition. High dietary fiber intake has been associated with a reduced risk of CRC, potentially through the production of short-chain fatty acids like butyrate, which possess anti-inflammatory and anti-neoplastic properties. Conversely, diets high in fat and low in fiber may promote the growth of harmful bacteria and increase bile acid secretion, contributing to CRC risk. Regular physical activity has also been shown to lower CRC risk, possibly by modulating immune function, reducing inflammation, and improving insulin sensitivity. Pharmacological interventions, particularly the use of glucagon-like peptide-1 receptor agonists (GLP-1 RAs) in DM2 management, have demonstrated potential in reducing CRC risk. Despite the high prevalence of obesity and metabolic comorbidities among CRC patients, studies investigating how lifestyle affects these conditions are limited. Advanced CRC patients experience malnutrition leading to weight loss, hypoglycemia, hypolipidemia, and reduced physical activity. Therefore, the complex interactions between lifestyle, obesity, metabolic comorbidities, and CRC are better explored in early-stage CRC patients.

The primary objective of our study was to evaluate the relationship between early-stage CRC patients’ lifestyle factors and the metabolic comorbidities such as overweight, obesity, MS, DM2 and MAFLD. The secondary objective of this study was to further investigate whether the aforementioned association differs by CRC onset age (early-onset vs. late-onset). Given the rapidly increasing incidence of early-onset CRC and the unclear underlying mechanisms, this study further investigates whether the association between lifestyle factors and metabolic comorbidities varies by CRC onset age, aiming to provide evidence for targeted interventions in age-specific populations.

## 2 Materials and methods

### 2.1 Study population

This is a multi-center cross-sectional study. The study population comprised patients who underwent curative colorectal endoscopic submucosal dissection (ESD) or endoscopic mucosal resection (EMR) for the management of early-stage CRC between April 2023 and March 2024 at the Second Hospital of Shandong University, Beijing Shijitan Hospital, and People’s Hospital of Xiajin County. The control group comprised asymptomatic individuals undergoing routine physical examinations at the same hospitals who had no family history of CRC and demonstrated no endoscopic evidence of colorectal polyps, malignant tumors, or inflammatory bowel disease on colonoscopy during the study period.

**Figure 1:**
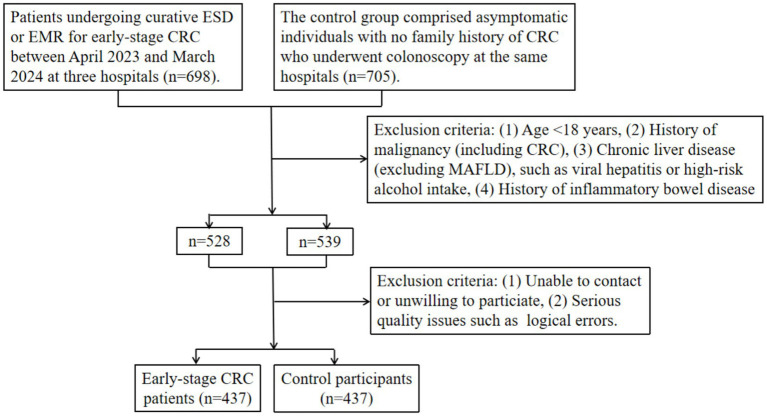
Flowchart of participant inclusion and exclusion criteria in the multi-center cross-sectional study. CRC: colorectal cancer. ESD: endoscopic submucosal dissection. EMR: endoscopic mucosal resection. MAFLD: metabolic dysfunction-associated fatty liver disease.

The exclusion criteria were as follows: (1) patients under 18 years old, (2) patients with the history of CRC or other malignant tumors, (3) patients with common causes of chronic liver disease other than MAFLD, such as viral hepatitis or high-risk alcohol intake (>140 g per week for women and > 210 g for men), (4) history of inflammatory bowel disease such as ulcerative colitis or Crohn’s disease, (5) patients without informed consent or with non-functional phone numbers, missing responses, or logical errors in the completed questionnaires (Figure 1).

The sample size for this study was calculated using the following formula (8):

$$n=\frac{Z^{2}P(1-P)}{d^{2}}$$

Z = 1.96 (*α* = 0.05), P = expected prevalence (based on the results of a preliminary study), d = 0.05 (expected prevalence ranges between 10 and 90%).

In our preliminary study, the prevalence of metabolic comorbidities in early-stage CRC patients and controls was as follows: MAFLD (28 and 23%), MS (21 and 17%), DM or prediabetes (25 and 20%), overweight (35 and 35%), and obesity (14 and 12%). Since higher expected prevalence necessitates larger samples, we selected overweight (35%) as the determinant for sample size estimation. Thus, the minimum required sample size is 350 participants. Accounting for 20% potential non-response, the target enrollment was 437. Thus, the final required sample size in our study comprised 437 early-stage CRC patients and 437 control participants.

### 2.2 Data collection

The study adopted a uniformly designed electronic data collection form, which covered all the clinical variables that needed to be extracted. The researchers at each center received standardized training to ensure the consistency of data definition. The data in the medical records were cross-checked by two independent researchers, and any discrepancies were resolved through arbitration by a third party. If there were missing or ambiguous information in the original records, follow-up calls were made to the patients or attending physicians for supplementary confirmation. To ensure the consistency of data from multiple centers, the research team held cross-center meetings monthly to verify the progress of data entry and address potential issues. In addition, all data were subjected to logical verification after being entered to exclude contradictory entries. The basic clinical data included sex, age, height, weight, smoking history, alcohol consumption history, medical history, family history, blood lipid level, and tumor characteristics (size, location, and pathology) of early-stage CRC. Body mass index (BMI) was calculated from the obtained height and weight using the formula: BMI = weight (kg)/ height (m^2^). BMI categories were defined as follows: underweight (<18 kg/m^2^), normal (18.0–23.9 kg/m^2^), overweight (24.0–27.9 kg/m^2^), and obese (≥28 kg/m^2^) (9).

### 2.3 Diagnostic criteria

The assessment of comorbidities was performed. The patients with fasting blood glucose ≥7.0 mmol/L or the glycated hemoglobin (HbA1c) level ≥6.5% were defined as DM2, whereas prediabetes was considered with fasting plasma glucose values ≥6.1 and <7.0 mmol/L or HbA1c between 5.7 and 6.4% (9). For MS, diagnosis was established by the presence of ≥3 factors: obesity/overweight, fasting blood glucose ≥6.1 mmol/L or treatment for diabetes, systolic/diastolic blood pressure ≥130/85 mmHg or treatment for hypertension, and fasting triglycerides ≥1.7 mmol/L or high density lipoprotein-cholesterol (HDL-C) < 1.04 mmol/L (9), and waist circumference (WC) ≥ 90 cm for men and ≥85 cm for women. MAFLD was diagnosed based on the presence of hepatic steatosis, detected by ultrasonography or a controlled attenuation parameter (CAP) > 248 dB/min, in addition to at least one of the following criteria: (1) overweight or obesity, (2) DM2, or (3) clinical evidence of metabolic dysfunction, defined as systolic/diastolic blood pressure ≥130/85 mmHg or use of specific antihypertensive medication, plasma triglycerides ≥1.7 mmol/L, or plasma HDL-C < 1.04 mmol/L in men or < 1.30 mmol/L in women (10).

Patients with advanced colorectal neoplasia or carcinoma including mucosal cancer and superficial submucosal cancer (invasion depth < 1,000 μm below the muscularis mucosae) were defined as early-stage CRC. Histopathologically, advanced colorectal neoplasia was characterized by polyps exhibiting at least 25% villous features, high-grade dysplasia, or a diameter ≥ 10 mm (11). For smoking status, ‘Yes’ indicated participants who self-reported current or historical tobacco consumption ≥ 7 cigarettes per week, and ‘No’ corresponded to those reporting consumption < 7 cigarettes per week or no tobacco use. For alcohol use status, ‘Yes’ indicated individuals reporting current or past drinking ≥ 2 times per week, and ‘No’ for those with consumption frequencies < 2 times per week or no alcohol use.

### 2.4 Diet and lifestyle

Dietary data included food composition, which was collected using the food frequency questionnaire constructed based on dietary habits and experiences in northern China. The food frequency questionnaire included 27 types of food, including rice, noodles, meat, fruits, vegetables, and nuts (Appendix A). We asked participants to recall food composition within the past year including diet frequency of each food (such as “never,” “per day,” “per week,” “per month,” or “per year”) and the average intake each time. Then we converted them into daily intake equivalents, and the dietary inflammatory index (DII) score for each participant was calculated based on the nutritional dietary information. DII was calculated according to method published previously (12). To calculate the DII score, 29 nutritional parameters, including protein, carbohydrates, cholesterol, energy, total fat, saturated fat, monounsaturated fatty acid, polyunsaturated fatty acid, omega-3, omega-6, trans fat, fiber, alcohol, caffeine, selenium, iron, magnesium, niacin, thiamin, riboflavin, folic acid, beta-carotene, vitamin A, vitamin B6, vitamin B12, vitamin C, vitamin D, vitamin E and zinc were considered. Through the completion of the Godin-Leisure Time Physical Activity Questionnaire, which was modified so that the sample exercises and sports provided for each category better matched the common activities of participants in northern China (Appendix B), the lifestyle of the patients was assessed. Before formally conducting the study, we collected data from 80 hospitalized patients who met the same inclusion and exclusion criteria as the formal survey. We conducted a test–retest reliability analysis of our questionnaires. Intraclass correlation coefficient (ICC) represented the measure of reliability. The food frequency questionnaire demonstrated good test–retest reliability (ICC = 0.879), and the Godin-Leisure Time Physical Activity Questionnaire also exhibited good reliability for both strenuous (ICC = 0.904) and moderate exercise (ICC = 0.873). We also conducted validity analyses of our questionnaires. The food frequency questionnaire demonstrated moderate criterion validity (Pearson’s *r* = 0.503), and the Godin-Leisure Time Physical Activity Questionnaire showed strong correlation coefficients for both strenuous (*r* = 0.741) and moderate exercise (*r* = 0.707) when validated against accelerometer data.

Definitions were established for the anti-inflammatory diet, active lifestyle, and healthy lifestyle. Specifically, a DII score < 0 was defined as an anti-inflammatory diet, while a DII score ≥ 0 was defined as an inflammatory diet. An active lifestyle was defined as a Godin-Leisure Time Physical Activity Questionnaire score ≥ 24 (13). Participants adhering to both an anti-inflammatory diet and an active lifestyle were classified as having a healthy lifestyle, while an unhealthy lifestyle was defined as failure to meet both criteria.

### 2.5 Statistical analysis

We described the basic features of the enrolled patients and control participants, including clinicopathological features and metabolic comorbidities conditions. Qualitative variables were displayed as numbers and percentages. The quantitative ones conformed to the normal distribution were displayed as means ± standard deviations (SDs), and quantitative variables conforming to non-normal distribution were displayed as medians + interquartile ranges (IQRs). We compared qualitative variables including basic features, overweight, obesity, metabolic comorbidities, dietary patterns, and lifestyle factors between early-stage CRC patients and controls using chi-square tests. We performed stratified analysis by age using the Cochran–Mantel–Haenszel test. Independent sample t test was further used to compare quantitative variables conforming to normally distribution, while Mann Whitney’s test was performed to compare quantitative variables conforming to non-normal distribution. All the variables that reached statistical significance in the univariate analysis were included in the multivariate analysis. A binomial generalized linear model with logistic regression analysis was used to study the impact of basic features and lifestyle factors on the existence of overweight, obesity, and comorbidities. Logistic regression analysis generated the odds ratio (OR) and 95% confidence interval (CI) for each factor. The variance inflation factor (VIF) was employed to assess multicollinearity among the variables using a threshold of VIF < 5. SPSS version 27 was used for performing the statistical analysis and *p*-value less than 0.05 was considered statistically significant.

## 3 Results

### 3.1 General characteristics

This study included 874 participants: 437 early-stage CRC patients and 437 control participants. The clinical characteristics, comorbidities, and dietary patterns were presented in Table 1. No significant differences were observed between the two groups in terms of age, sex distribution, smoking history, metabolic comorbidities, lifestyle factors and so on (*p* > 0.05). The median age of participants was 63 (IQR 51–71) and 64 (IQR 54–72) years in the control and early-stage CRC groups, respectively. The proportion of males were higher in the both groups. Early-stage CRC patients exhibited higher prevalence of MS (21.74% vs. 17.16%), DM2 or prediabetes (24.49% vs. 20.59%), and MAFLD (28.15% vs. 24.26%) than controls, though these differences were not statistically significant (*p* > 0.05). An anti-inflammatory diet (42.79% vs. 48.74%), an active lifestyle (30.21% vs. 33.41%), and a healthy lifestyle (17.85% vs. 18.99%) were less frequently observed in early-stage CRC patients than in controls, with all intergroup differences non-significant (*p* > 0.05). Among early-stage CRC patients, pathological evaluation revealed carcinoma in 59 patients (13.50%) and advanced colorectal neoplasia in 378 patients (86.50%).

**Table 1:** General characteristics of the early-stage CRC patients and control participants included in the research.

Variables	Units/Categories	Control (*n* = 437)	Early-stage CRC (*n* = 437)	p*	Variables	Units/Categories	Control (*n* = 437)	Early-stage CRC (*n* = 437)	p*
Age, median (IQR)	Years	63 (51–71)	64 (54–72)	0.245	Waist circumference, median (IQR)	Cm	82.60 (78.85–87.45)	83.20 (80.30–87.15)	0.060
Gender, *n* (%)	Male	251 (57.44)	275 (62.93)	0.097	High total cholesterol, *n* (%)	Yes	20 (4.58)	31 (7.09)	0.112
	Female	186 (42.56)	162 (37.07)			No	417 (95.42)	406 (92.91)	
Smoking, *n* (%)	Yes	112 (25.63)	131 (29.98)	0.151	Low HDL-C, *n* (%)	Yes	31 (7.09)	39 (8.92)	0.319
	No	325 (74.37)	306 (70.02)			No	406 (92.91)	398 (91.08)	
Alcohol, *n* (%)	Yes	131 (29.98)	156 (35.70)	0.072	High LDL-C, *n* (%)	Yes	44 (10.07)	55 (12.59)	0.240
	No	306 (70.02)	281 (64.30)			No	393 (89.93)	382 (87.41)	
Body mass index, *n* (%)	Low weight	12 (2.75)	5 (1.15)	0.119	High triglycerides, *n* (%)	Yes	49 (11.21)	58 (13.27)	0.353
	Normal weight	215 (49.20)	194 (44.39)			No	388 (88.79)	379 (86.73)	
	Overweight	156 (35.70)	173 (39.59)		Location, *n* (%)	Proximal colon	-	229 (52.40)	-
	Obesity	54 (12.35)	65 (14.87)			Distal colon	-	156 (35.70)	-
DM2 or prediabetes, *n* (%)	Yes	90 (20.59)	107 (24.49)	0.169		Rectum	-	52 (11.90)	-
	No	347 (79.41)	330 (75.51)		Size, *n* (%)	<10 mm	-	79 (18.08)	-
Cardiovascular disease, *n* (%)	Yes	61 (13.96)	74 (16.93)	0.224		≥10 mm	-	358 (81.92)	-
	No	376 (86.04)	363 (83.07)		Pathology, *n* (%)	Carcinoma	-	59 (13.50)	-
Cerebrovascular disease, *n* (%)	Yes	28 (6.41)	37 (8.47)	0.246		Advanced colorectal neoplasia	-	378 (86.50)	-
	No	409 (93.59)	400 (91.53)		Anti-inflammatory diet, *n* (%)	Yes	213 (48.74)	187 (42.79)	0.078
Hypertension, *n* (%)	Yes	129 (29.52)	153 (35.01)	0.082		No	224 (51.26)	250 (57.21)	
	No	308 (70.48)	284 (64.99)		Active lifestyle, *n* (%)	Yes	146 (33.41)	132 (30.21)	0.309
Metabolic syndrome, *n* (%)	Yes	75 (17.16)	95 (21.74)	0.087		No	291 (66.59)	305 (69.79)	
	No	362 (82.84)	342 (78.26)		Healthy lifestyle, *n* (%)	Yes	83 (18.99)	78 (17.85)	0.663
MAFLD, *n* (%)	Yes	106 (24.26)	123 (28.15)	0.191		No	354 (81.01)	359 (82.15)	
	No	331 (75.74)	314 (71.85)						

CRC: colorectal cancer. IQR: interquartile range. DM2: type 2 diabetes mellitus. MAFLD: metabolic dysfunction-associated fatty liver disease. HDL-C: high density lipoprotein-cholesterol. LDL-C: low density lipoprotein-cholesterol. Smoking, Yes indicated current or past smokers (≥7 cigarettes/week), and No indicated non-smokers (<7 cigarettes/week) or no tobacco use. Alcohol, Yes indicated current or past drinkers (≥2 times/week), and No indicated non-drinkers (<2 times/week) or no alcohol use. The potential confounding effects of lipid-lowering therapies were not accounted for due to unavailable pharmacotherapy records. p*, Control vs. early-stage CRC, *p* < 0.05 was considered as statistically significant.

### 3.2 Impact of lifestyle factors on overweight and obesity in early-stage CRC patients

In univariate analyses, overweight and obesity were significantly associated with older age, male sex, an anti-inflammatory diet, an active lifestyle, and a healthy lifestyle. In addition, smoking was significantly associated with obesity (*p* < 0.05; Table 2). Multivariate analyses revealed that older age (OR = 1.043, 95% CI = 1.024–1.063, *p* < 0.001) and an anti-inflammatory diet (OR = 0.585, 95% CI = 0.346–0.988, *p* = 0.045) were independently associated with overweight. Obesity was associated with older age (OR = 1.027, 95% CI = 1.002–1.053, *p* = 0.034), male sex (OR = 2.125, 95% CI = 1.080–4.182, *p* = 0.029), an anti-inflammatory diet (OR = 0.463, 95% CI = 0.221–0.966, *p* = 0.040), and smoking (OR = 1.898, 95% CI = 1.016–3.546, *p* = 0.044). In contrast, among control participants, significant associations were observed only with older age and male sex (*p* < 0.05; Supplementary Tables S1, S2). All multivariate analyses demonstrated that VIF values ranged from 1.009 to 3.144 (all values < 5), indicating no significant multicollinearity among the variables.

**Table 2:** Univariate analyses results: the association between lifestyle factors and overweight/obesity in early-stage CRC patients.

Variables	Units/Categories	No Overweight/Obesity (*n* = 199)	Overweight (*n* = 173)	p*	Obesity (*n* = 65)	*p*#
Age, median (IQR)	Years	61 (51–68)	67 (58–73)	<0.001	66 (52.5–76)	0.019
Gender, *n* (%)	Male	111 (55.78)	116 (67.05)	0.026	48 (73.85)	0.01
	Female	88 (44.22)	57 (32.95)		17 (26.15)	
Smoking, *n* (%)	Yes	50 (25.13)	55 (31.79)	0.154	26 (40)	0.021
	No	149 (74.87)	118 (68.21)		39 (60)	
Alcohol, *n* (%)	Yes	62 (31.16)	67 (38.73)	0.126	27 (41.54)	0.124
	No	137 (68.84)	106 (61.27)		38 (58.46)	
Location, *n* (%)	Proximal colon	105 (52.76)	88 (50.87)	0.798	36 (55.38)	0.932
	Distal colon	72 (36.18)	62 (35.84)		22 (33.85)	
	Rectum	22 (11.06)	23 (13.29)		7 (10.77)	
Size, *n* (%)	<10 mm	43 (21.61)	25 (14.45)	0.075	11 (16.92)	0.416
	≥10 mm	156 (78.39)	148 (85.55)		54 (83.08)	
Pathology, *n* (%)	Carcinoma	24 (12.06)	22 (12.72)	0.848	13 (20.00)	0.109
	Advanced colorectal neoplasia	175 (87.94)	151 (87.28)		52 (80.00)	
Anti-inflammatory diet, *n* (%)	Yes	102 (51.26)	65 (37.57)	0.008	20 (30.77)	0.004
	No	97 (48.74)	108 (62.43)		45 (69.23)	
Active lifestyle, *n* (%)	Yes	73 (36.68)	45 (26.01)	0.027	14 (21.54)	0.024
	No	126 (63.32)	128 (73.99)		51 (78.46)	
Healthy lifestyle, *n* (%)	Yes	47 (23.62)	24 (13.87)	0.017	7 (10.77)	0.026
	No	152 (76.38)	149 (86.13)		58 (89.23)	

CRC: colorectal cancer. IQR: interquartile range. Smoking, Yes indicated current or past smokers (≥ 7 cigarettes/week), and No indicated non-smokers (<7 cigarettes/week) or no tobacco use. Alcohol, Yes indicated current or past drinkers (≥2 times/week), and No indicated non-drinkers (<2 times/week) or no alcohol use. p*, Overweight vs. No Overweight/Obesity; p#, Obesity vs. No Overweight/Obesity; *p* < 0.05 was considered as statistically significant.

### 3.3 Impact of lifestyle factors on metabolic comorbidities in early-stage CRC patients

In univariate analyses, all these comorbidities showed significant associations with older age, male sex, smoking, overweight or obesity, an inactive lifestyle, and an unhealthy lifestyle (*p* < 0.05; Table 3). Multivariate analyses demonstrated that MS was statistically associated with older age (OR = 1.052, 95% CI = 1.030–1.073, *p* < 0.001) and overweight or obesity (OR = 2.203, 95% CI = 1.283–3.782, *p* = 0.004). Similarly, DM2 or prediabetes was significantly associated with older age (OR = 1.053, 95% CI = 1.032–1.073, *p* < 0.001) and overweight or obesity (OR = 1.788, 95% CI = 1.079–2.960, *p* = 0.024). MAFLD was significantly associated with older age (OR = 1.039, 95% CI = 1.020–1.058, *p* < 0.001), overweight or obesity (OR = 1.807, 95% CI = 1.122–2.910, *p* = 0.015), and an unhealthy lifestyle (OR = 4.314, 95% CI = 1.549–12.014, *p* = 0.005) (Table 4). In contrast, associations in control participants were limited to older age, male sex, and overweight or obesity (*p* < 0.05; Supplementary Tables S3, S4). All multivariate analyses demonstrated VIF values ranged from 1.022 to 2.182 (all values < 5), indicating no significant multicollinearity among the variables.

**Table 3:** Univariate analyses results: the association between lifestyle factors and metabolic comorbidities in early-stage CRC patients.

Variables	Units/Categories	Metabolic syndrome		p*	DM2 or prediabetes		*p*#	MAFLD		p&
		Yes (*n* = 95)	No (*n* = 342)		Yes (*n* = 107)	No (*n* = 330)		Yes (*n* = 123)	No (*n* = 314)	
Age, median (IQR)	Years	71 (64–75)	62 (52.75–70)	<0.001	70 (64–75)	62 (52–70)	<0.001	69 (61–75)	62 (52–70)	<0.001
Gender, *n* (%)	Male	68 (71.58)	207 (60.53)	0.048	76 (71.03)	199 (60.30)	0.046	87 (70.73)	188 (59.87)	0.035
	Female	27 (28.42)	135 (39.47)		31 (28.97)	131 (39.70)		36 (29.27)	126 (40.13)	
Smoking, *n* (%)	Yes	37 (38.95)	94 (27.49)	0.031	41 (38.32)	90 (27.27)	0.030	48 (39.02)	83 (26.43)	0.010
	No	58 (61.05)	248 (72.51)		66 (61.68)	240 (72.73)		75 (60.98)	231 (73.57)	
Alcohol, *n* (%)	Yes	34 (35.79)	122 (35.67)	0.983	37 (34.58)	119 (36.06)	0.781	48 (39.02)	108 (34.39)	0.364
	No	61 (64.21)	220 (64.33)		70 (65.42)	211 (63.94)		75 (60.98)	206 (65.61)	
Overweight or obesity, *n* (%)	Yes	71 (74.74)	167 (48.83)	<0.001	76 (71.03)	162 (49.09)	<0.001	86 (69.92)	152 (48.41)	<0.001
	No	24 (25.26)	175 (51.17)		31 (28.97)	168 (50.91)		37 (30.08)	162 (51.59)	
Location, *n* (%)	Proximal colon	47 (49.47)	182 (53.22)	0.753	55 (51.40)	174 (52.73)	0.972	60 (48.78)	169 (53.82)	0.462
	Distal colon	35 (36.84)	121 (35.38)		39 (36.45)	117 (35.45)		45 (36.59)	111 (35.35)	
	Rectum	13 (13.69)	39 (11.40)		13 (12.15)	39 (11.82)		18 (14.63)	34 (10.83)	
Size, *n* (%)	<10 mm	18 (18.95)	61 (17.84)	0.803	20 (18.69)	59 (17.88)	0.849	23 (18.70)	56 (17.83)	0.833
	≥10 mm	77 (81.05)	281 (82.16)		87 (81.31)	271 (82.12)		100 (81.30)	258 (82.17)	
Pathology, *n* (%)	Carcinoma	18 (18.95)	41 (11.99)	0.079	20 (18.69)	39 (11.82)	0.071	21 (17.07)	38 (12.10)	0.171
	Advanced colorectal neoplasia	77 (81.05)	301 (88.01)		87 (81.31)	291 (88.18)		102 (82.93)	276 (87.90)	
Anti-inflammatory diet, *n* (%)	Yes	33 (34.74)	154 (45.03)	0.073	38 (35.51)	149 (45.15)	0.08	45 (36.59)	142 (45.22)	0.101
	No	62 (65.26)	188 (54.97)		69 (64.49)	181 (54.85)		78 (63.41)	172 (54.78)	
Active lifestyle, *n* (%)	Yes	18 (18.95)	114 (33.33)	0.007	21 (19.63)	111 (33.64)	0.006	24 (19.51)	108 (34.39)	0.002
	No	77 (81.05)	228 (66.67)		86 (80.37)	219 (66.36)		99 (80.49)	206 (65.61)	
Healthy lifestyle, *n* (%)	Yes	9 (9.47)	69 (20.18)	0.016	11 (10.28)	67 (20.30)	0.019	7 (5.69)	71 (22.61)	<0.001
	No	86 (90.53)	273 (79.82)		96 (89.72)	263 (79.70)		116 (94.31)	243 (77.39)	

CRC: colorectal cancer. DM2: type 2 diabetes mellitus. MAFLD: metabolic dysfunction-associated fatty liver disease. IQR: interquartile range. Smoking, Yes indicated current or past smokers (≥7 cigarettes/week), and No indicated non-smokers (<7 cigarettes/week) or no tobacco use. Alcohol, Yes indicated current or past drinkers (≥2 times/week), and No indicated non-drinkers (<2 times/week) or no alcohol use. p*, Metabolic Syndrome, Yes vs. No; p#, DM2 or prediabetes, Yes vs. No; p&, MAFLD, Yes vs. No; *p* < 0.05 was considered as statistically significant.

**Table 4:** Logistic regression results: the association between lifestyle factors and metabolic comorbidities in early-stage CRC patients.

Variables	Metabolic syndrome			DM2 or prediabetes			MAFLD		
	OR	95% CI	*p*	OR	95% CI	*p*	OR	95% CI	*p*
Age	1.052	1.030–1.073	<0.001	1.053	1.032–1.073	<0.001	1.039	1.020–1.058	<0.001
Male sex	0.827	0.468–1.460	0.512	0.802	0.467–1.379	0.425	0.964	0.581–1.600	0.887
Smoking	0.702	0.421–1.168	0.173	0.698	0.427–1.141	0.151	0.663	0.415–1.059	0.086
Overweight or obesity	2.203	1.283–3.782	0.004	1.788	1.079–2.960	0.024	1.807	1.122–2.910	0.015
Inactive lifestyle	1.768	0.792–3.947	0.164	1.812	0.839–3.914	0.130	1.025	0.533–1.972	0.940
Unhealthy lifestyle	1.161	0.390–3.455	0.788	1.070	0.385–2.970	0.897	4.314	1.549–12.014	0.005

CRC: colorectal cancer. DM2: type 2 diabetes mellitus. MAFLD: metabolic dysfunction-associated fatty liver disease. OR: odds ratio. CI: confidence interval. *p* < 0.05 was considered as statistically significant.

**Figure 2:**
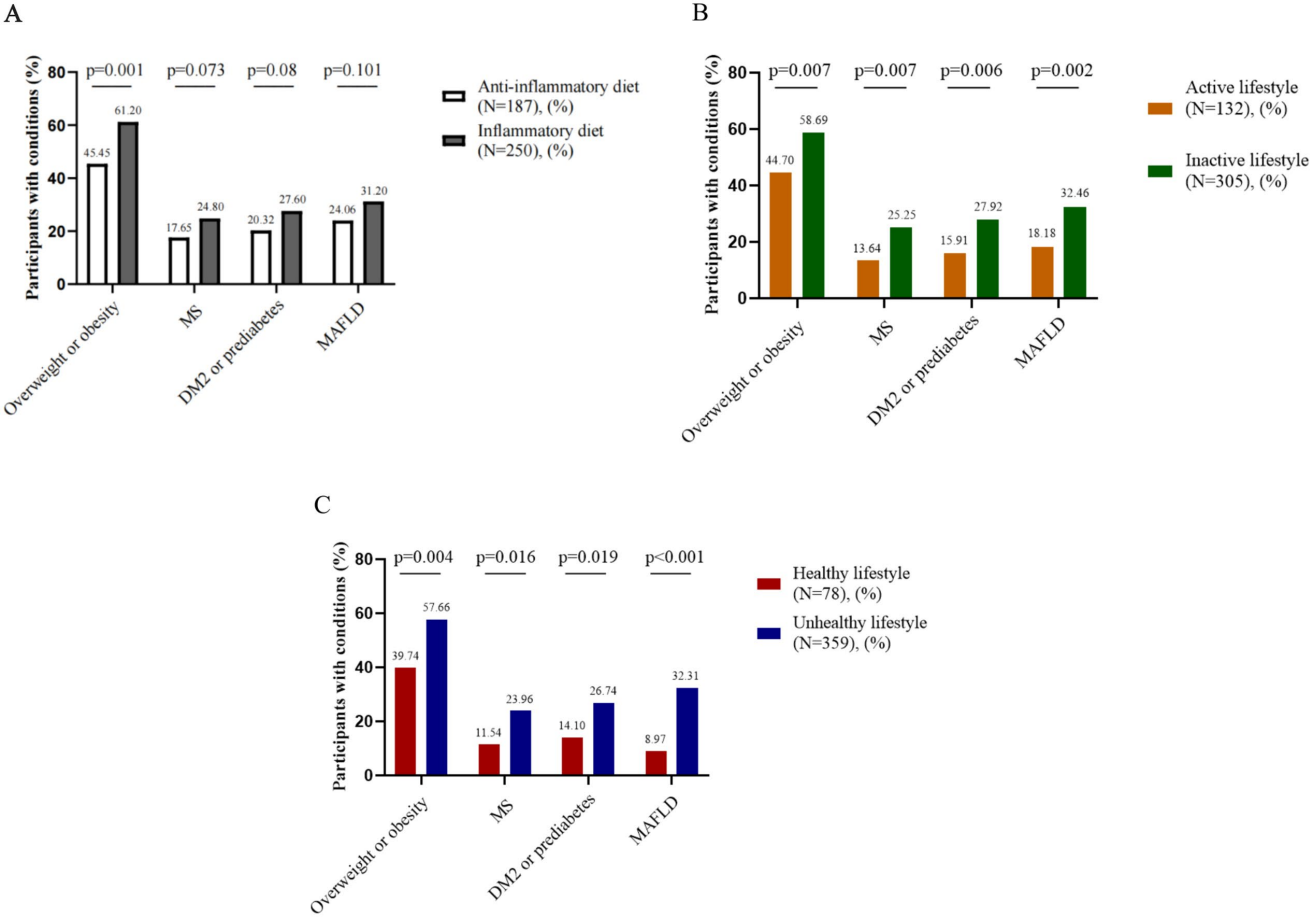
Associations between lifestyle factors and metabolic comorbidities in early-stage CRC patients. **(A)** Anti-inflammatory diet and metabolic comorbidities, **(B)** Active lifestyle and metabolic comorbidities, **(C)** Healthy lifestyle and metabolic comorbidities. CRC: colorectal cancer. MS: metabolic syndrome. DM2: type 2 diabetes mellitus. MAFLD: metabolic dysfunction-associated fatty liver disease.

In this study, we investigated the associations of an anti-inflammatory diet, an active lifestyle and a healthy lifestyle with the diagnosis of overweight or obesity, MS, DM2 or prediabetes, and MAFLD. In early-stage CRC patients, adherence to an anti-inflammatory diet was significantly associated with a lower likelihood of being diagnosed with overweight or obesity (*p* < 0.05), with potential associations observed for MS, DM2 or prediabetes, and MAFLD. Additionally, both an active lifestyle and a healthy lifestyle were significantly associated with reduced odds of overweight or obesity, MS, DM2 or prediabetes, and MAFLD (*p* < 0.05) (Figure 2). In the control participants, adherence to an anti-inflammatory diet, an active lifestyle, or a healthy lifestyle was not significantly associated with a diagnosis of overweight or obesity, MS, DM2 or prediabetes, or MAFLD (Supplementary Figure S1).

### 3.4 Stratified analyses of associations between lifestyle factors and metabolic comorbidities in early-onset vs. late-onset CRC

Stratified analyses revealed that smokers with late-onset CRC showed significantly higher risks of developing MS (OR = 1.718, 95% CI = 1.031–2.862, *p* = 0.037), DM2 or prediabetes (OR = 1.753, 95% CI = 1.070–2.872, *p* = 0.025), and MAFLD (OR = 1.785, 95% CI = 1.107–2.878, *p* = 0.017). Conversely, patients adhering to an active lifestyle and a healthy lifestyle demonstrated risk reductions for these metabolic comorbidities (*p* < 0.05). Notably, these associations were absent in the early-onset CRC cohort (*p* > 0.05; Table 5).

**Table 5:** Stratified analyses of associations between lifestyle factors and metabolic comorbidities in early-onset vs. late-onset CRC.

Stratification	Smoking, *n* (%)	Metabolic syndrome		OR	95% CI	*p*	DM2 or prediabetes		OR	95% CI	*p*	MAFLD		OR	95% CI	*p*
		Yes (*n* = 95)	No (*n* = 342)				Yes (*n* = 107)	No (*n* = 330)				Yes (*n* = 123)	No (*n* = 314)			
Early-onset	Yes	3 (27.27)	18 (24.32)	1.167	0.279–4.871	1	3 (23.08)	18 (25.00)	0.900	0.223–3.636	1	5 (31.25)	16 (23.19)	1.506	0.455–4.978	0.725
	No	8 (72.73)	56 (75.68)				10 (76.92)	54 (75.00)				11 (68.75)	53 (76.81)			
Late-onset	Yes	34 (40.48)	76 (28.36)	1.718	1.031–2.862	0.037	38 (40.43)	72 (27.91)	1.753	1.070–2.872	0.025	43 (40.19)	67 (27.35)	1.785	1.107–2.878	0.017
	No	50 (59.52)	192 (71.64)				56 (59.57)	186 (72.09)				64 (59.81)	178 (72.65)			
	Active lifestyle, n (%)															
Early-onset	Yes	1 (9.09)	17 (22.97)	0.335	0.040–2.810	0.512	2 (15.38)	16 (22.22)	0.636	0.128–3.170	0.852	4 (25.00)	14 (20.29)	1.310	0.366–4.685	0.939
	No	10 (90.91)	57 (77.03)				11 (84.62)	56 (77.78)				12 (75.00)	55 (79.71)			
Late-onset	Yes	17 (20.24)	97 (36.19)	0.447	0.249–0.805	0.006	19 (20.21)	95 (36.82)	0.435	0.247–0.764	0.003	20 (18.69)	94 (38.37)	0.369	0.213–0.640	< 0.001
	No	67 (79.76)	171 (63.81)				75 (79.79)	163 (63.18)				87 (81.31)	151 (61.63)			
	Healthy lifestyle, *n* (%)															
Early-onset	Yes	0 (0)	8 (10.81)	0.514	0.141–2.902	0.554	1 (7.69)	7 (9.72)	0.774	0.087–6.872	1	2 (12.50)	6 (8.70)	1.5	0.274–8.226	1
	No	11 (100)	66 (89.19)				12 (92.31)	65 (90.28)				14 (87.50)	63 (91.30)			
Late-onset	Yes	9 (10.71)	61 (22.76)	0.407	0.193–0.860	0.016	10 (10.64)	60 (23.26)	0.393	0.192–0.804	0.009	5 (4.67)	65 (26.53)	0.136	0.053–0.348	< 0.001
	No	75 (89.29)	207 (77.24)				84 (89.36)	198 (76.74)				102 (95.33)	180 (73.47)			

CRC: colorectal cancer. DM2: type 2 diabetes mellitus. MAFLD: metabolic dysfunction-associated fatty liver disease. OR: odds ratio. CI: confidence interval. Smoking, Yes indicated current or past smokers (≥7 cigarettes/week), and No indicated non-smokers (<7 cigarettes/week) or no tobacco use. *p* < 0.05 was considered as statistically significant.

## 4 Discussion

The global prevalences of obesity and obesity-associated metabolic comorbidities, including MS, DM2, and MAFLD, are on the rise (14, 15), which is consistent with the CRC incidence trend.

In our study, overweight and obesity among early-stage CRC patients, respectively, accounted for 39.59 and 14.87%, which were slightly lower than the findings from the Polish reported researches with overweight rates of 44.53% and obesity rates of 27.66%, respectively (16). Among early-stage CRC patients, male sex was an independent risk factor for obesity (OR = 2.125, 95% CI = 1.080–4.182, *p* = 0.029), and increasing age was an independent risk factor for both overweight (OR = 1.043, 95% CI = 1.024–1.063, *p* < 0.001) and obesity (OR = 1.027, 95% CI = 1.002–1.053, *p* = 0.034), but these associations were also observed among control participants. Estrogen may play a protective role against weight gain and obesity (17). In addition, differences in fat distribution patterns (visceral accumulation in men vs. gluteal and femoral accumulation in women) represent another important factor. Previous studies have documented an age-related increase in the prevalence of overweight and obesity (18). The mechanisms linking aging to obesity are complex. Aging is associated with diminished brown adipose tissue activity, leptin resistant, insulin resistant, and chronic systemic inflammation (19). These factors contribute to energy imbalance and obesity. Furthermore, aging increases the risks of obesity-associated complications, including MS and DM2 (20–22). Age-dependent changes in adipose tissue, characterized by altered distribution, impaired adipokine secretion, reduced adipogenesis, and increased lipotoxicity, significantly contribute to the pathogenesis of these obesity-related complications. Among early-stage CRC patients, aging was associated not only with overweight and obesity but also with the development of MS (OR = 1.052, 95% CI = 1.030–1.073, *p* < 0.001), DM2 or prediabetes (OR = 1.053, 95% CI = 1.032–1.073, *p* < 0.001), and MAFLD (OR = 1.039, 95% CI = 1.020–1.058, *p* < 0.001), as observed among controls. Moreover, smoking was also an independent risk factor for obesity (OR = 1.898, 95% CI = 1.016–3.546, *p* = 0.044) among early-stage CRC patients, suggesting that smoking may exacerbate metabolic dysregulation in this population. These findings are consistent with previous reports demonstrating that smoking increases the risk of overweight and obesity (23). Our findings may help explain the association between smoking and obesity-related complications. However, we found no association between overweight or obesity and tumor location in early-stage CRC patients as described in previous studies (24). Among early-stage CRC patients, overweight and obesity represented an independent risk factor for developing MS (OR = 2.203, 95% CI = 1.283–3.782, *p* = 0.004), DM2 or prediabetes (OR = 1.788, 95% CI = 1.079–2.960, *p* = 0.024), and MAFLD (OR = 1.807, 95% CI = 1.122–2.910, *p* = 0.015). Although these factors may explain the development of overweight or obesity and metabolic comorbidities in early-stage CRC, our results indicated that lifestyle factors also play a critical role.

We found that an anti-inflammatory diet (DII score < 0) was associated with a lower likelihood of overweight (OR = 0.585, 95% CI = 0.346–0.988, *p* = 0.045) and obesity (OR = 0.463, 95% CI = 0.221–0.966, *p* = 0.040) among early-stage CRC patients, but this association was not observed in control participants. The DII assesses the inflammatory potential of diets, worldwide and predicts serum inflammatory factor levels and the development of low-grade, chronic systemic inflammation (12). Previous studies with varying designs support the protective role of anti-inflammatory diets against metabolic disease (25–27). Although not statistically significant, diagnosis of MAFLD, DM2 or prediabetes, and MS occurred more frequently among early-stage CRC patients who did not adhere to an anti-inflammatory diet.

Although the relationship between metabolic diseases and CRC gets much attention and a large number of studies have confirmed that metabolic disease was linked to an increased risk of CRC incidence (28–30), the influence of lifestyle factors in CRC patients on metabolic diseases is less studied. Among early-stage CRC patients, an active lifestyle and a healthy lifestyle (defined as adherence to both an anti-inflammatory diet and active physical activity) were associated with significantly lower prevalence of overweight or obesity, MS, DM2 or prediabetes, and MAFLD. Conversely, an unhealthy lifestyle (OR = 4.314, 95% CI = 1.549–12.014, *p* = 0.005) was independently associated with MAFLD development. These findings suggested that inactive lifestyle and unhealthy lifestyles in early-stage CRC patients were associated with increased risk of metabolic diseases. Physical inactivity is increasingly recognized to adversely influence metabolic disorders such as DM2, MAFLD through multiple interrelated mechanisms. Chronic low-grade inflammation appears to be a key pathway: sedentary behavior is associated with elevated proinflammatory mediators, and a vicious cycle can develop wherein physical inactivity, obesity, and inflammation exacerbate one another (31, 32). This in turn contributes to metabolic disorders like DM2 and MAFLD (33). In addition, lack of exercise may unfavorably alter the gut microbiota and intestinal environment. By contrast, regular physical activity has been shown to increase anti-inflammatory mediators and improve gut barrier function. Intervention studies in MS cohorts have shown modest improvements. For example, a 6-month trial of sedentary-time reduction lowered fasting insulin but did not significantly improve whole-body insulin sensitivity, suggesting that more intensive or combined lifestyle interventions including healthy dietary habits and weight loss might be required to confer significant metabolic benefits (34). Moreover, although the overall recurrence-free survival rate for early-stage CRC exceeds 95% (35), the long-term risk of metachronous neoplasia persists (36). Given that MS and MAFLD are reversible, lifestyle interventions in such patients represent a potential strategy for preventing metachronous neoplasia after radical resection, which needs to be further investigated.

The stratified analysis findings suggest notable differences in the impact of lifestyle factors between early-onset and late-onset CRC cohorts. Specifically, smoking was significantly associated with an elevated risk of MS, DM2 or prediabetes, and MAFLD exclusively in late-onset CRC patients. These findings align with existing literature demonstrating smoking as an independent risk factor exacerbating metabolic disorders, particularly in older populations, due to chronic inflammation and insulin resistance pathways (37). Conversely, adherence to an active and healthy lifestyle markedly reduced the risk of these metabolic comorbidities in late-onset CRC patients, reinforcing the protective effects of physical activity and anti-inflammatory dietary patterns through mitigation of chronic inflammation, improved insulin sensitivity, and enhanced gut barrier function (31, 33, 34). Interestingly, these associations were not observed in early-onset CRC patients, which might be explained by differences in pathogenesis, disease progression, and distinct lifestyle patterns typically seen among younger populations. Previous studies suggest younger CRC patients often present distinct genetic predispositions or unique microbiota profiles, potentially overshadowing the impacts of lifestyle factors observed in older cohorts (38, 39). This disparity emphasizes the need for tailored, age-specific lifestyle interventions in the management of CRC and related metabolic disorders.

It is important to highlight several strengths in this study. First, our study is a multi-center analysis from three different hospitals. Second, to the best of our knowledge, there have been limited studies investigating the association between lifestyle factors and early-stage CRC. Previous researches have predominantly focused on examining the impact of lifestyle on colorectal cancer risk in general populations (40–42). Our study including 437 early-stage CRC patients and 437 control participants is the one of the largest cross-sectional cohort study that examines the association between lifestyle factors, including adherence to an anti-inflammatory diet and physical exercise, and prevalent comorbidities (MS, DM2, and MAFLD) in early-stage CRC patients. Third, we included early-stage CRC patients to examine the association between lifestyle factors and metabolic comorbidities. Cachexia and malnutrition in advanced CRC induce systemic inflammation and altered metabolism independent of lifestyle. Focusing on early-stage patients minimizes these confounding effects, clarifying direct associations between lifestyle factors and metabolic comorbidities.

There are several limitations in our study. First, this study is constrained by its cross-sectional design. The causal relationships between lifestyle factors and metabolic comorbidities in early-stage CRC patients are difficult to establish. Second, there may be information bias in questionnaire survey. The data collected from the questionnaire survey were based on patient self-reports rather than being objectively observed by an external evaluator. Furthermore, socioeconomic status factors such as educational attainment, income level, and occupational category may serve as potential confounding variables influencing adherence to lifestyle modifications. The smoking data were categorized into a binary classification (yes/no), without detailed information on cumulative exposure or cessation history. Future longitudinal studies should confirm whether the observed associations reflect protective effects of healthy lifestyles against metabolic comorbidities over time, investigate the effects of specific dietary patterns on metabolic comorbidities in early-stage CRC patients, and incorporate comprehensive smoking history data to explore dose–response relationships, thereby overcoming current limitations. Furthermore, future research should integrate microbiota analyses through fecal sampling to characterize dysbiosis patterns and inflammatory responses, potentially revealing mechanistic links between diet, lifestyle, and metabolic disorders in CRC. Stratified studies across various CRC stages would also help delineate precise interactions among lifestyle factors, diet, microbiota, and metabolic comorbidities, enabling tailored and stage-specific therapeutic approaches.

## 5 Conclusion

These findings suggest that adherence to healthy lifestyles, particularly in individuals aged ≥50 years, may represent a potential strategy for ameliorating metabolic dysregulation in early-stage CRC patients. Further prospective studies are needed to confirm these observational associations and clarify their clinical implications.

## Data Availability

The raw data supporting the conclusions of this article will be made available by the authors, without undue reservation.
