# Supplementary material for: Lifestyle can exert a significant impact on the development of metabolic comorbidities in early-stage colorectal cancer patients
Source: Front Nutr. 2025 Jul 4;12:1551526. doi: 10.3389/fnut.2025.1551526 (PMC12272228; doi:10.3389/fnut.2025.1551526)
Supplement: Supplementary file 1 [file Data_Sheet_1.docx]

A


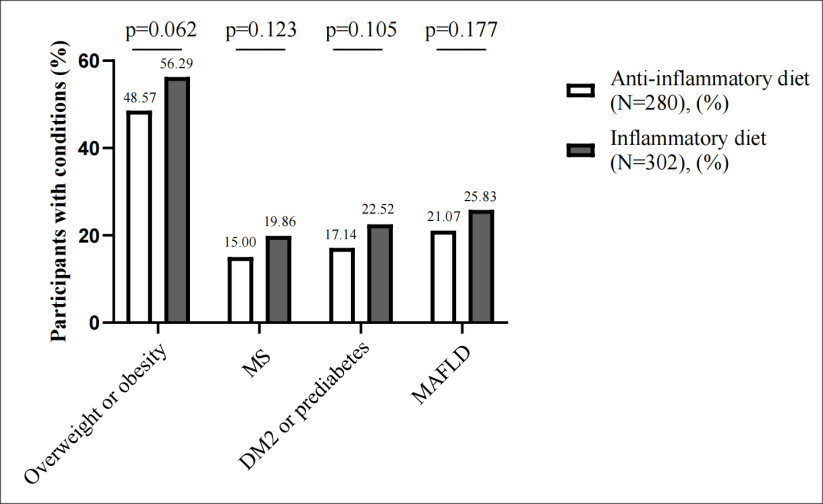


B


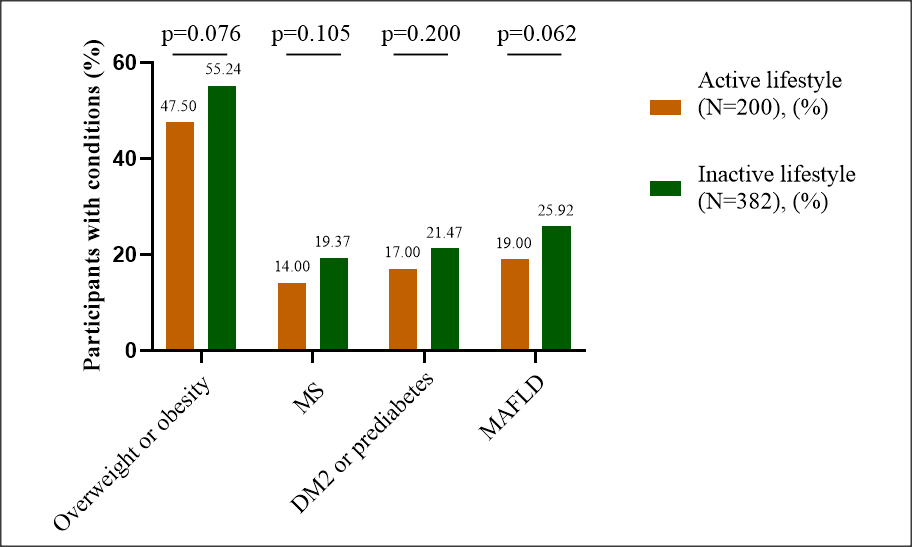


C


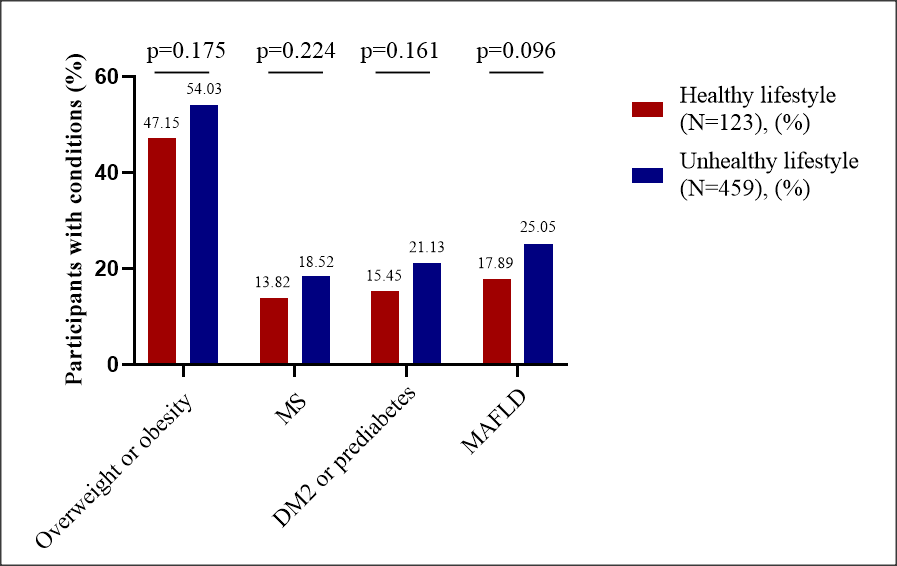


Figure S1. Associations between lifestyle factors and metabolic comorbidities in control participants. A, Anti-inflammatory diet and metabolic comorbidities, B, Active lifestyle and metabolic comorbidities, C, Healthy lifestyle and metabolic comorbidities. CRC: colorectal cancer. MS: metabolic syndrome. DM2: type 2 diabetes mellitus. MAFLD: metabolic dysfunction-associated fatty liver disease.
