# Supplementary material for: Lifestyle can exert a significant impact on the development of metabolic comorbidities in early-stage colorectal cancer patients
Source: Front Nutr. 2025 Jul 4;12:1551526. doi: 10.3389/fnut.2025.1551526 (PMC12272228; doi:10.3389/fnut.2025.1551526)
Supplement: Supplementary file 2 [file Data_Sheet_2.docx]

Appendix A: Food Frequency Questionnaire

Please recall whether you have consumed the following foods in the past year and estimate their average consumption quantity and frequency.

| Foods Category | Average consumption per serving | Eating frequency | | | | |
| --- | --- | --- | --- | --- | --- | --- |
|  |  | Daily | Weekly | Monthly | Yearly | Not consumed |
|  | gram | Please select an appropriate time period and write the appropriate frequency | | | | |
| Rice |  |  |  |  |  |  |
| Steamed Bun (Mantou) |  |  |  |  |  |  |
| Millet |  |  |  |  |  |  |
| Noodles |  |  |  |  |  |  |
| Fried Dough Stick (Youtiao) |  |  |  |  |  |  |
| Tofu |  |  |  |  |  |  |
| Pork |  |  |  |  |  |  |
| Pork Ribs |  |  |  |  |  |  |
| Beef |  |  |  |  |  |  |
| Mutton |  |  |  |  |  |  |
| Chicken |  |  |  |  |  |  |
| Fish |  |  |  |  |  |  |
| Shrimp |  |  |  |  |  |  |
| Chicken Egg |  |  |  |  |  |  |
| Milk |  |  |  |  |  |  |
| Yogurt |  |  |  |  |  |  |
| Soy Milk |  |  |  |  |  |  |
| Apple |  |  |  |  |  |  |
| Pear |  |  |  |  |  |  |
| Banana |  |  |  |  |  |  |
| Spinach |  |  |  |  |  |  |
| Potato |  |  |  |  |  |  |
| Cauliflower |  |  |  |  |  |  |
| Chinese Cabbage |  |  |  |  |  |  |
| Tomato |  |  |  |  |  |  |
| Peanut |  |  |  |  |  |  |
| Pistachio |  |  |  |  |  |  |
