# Supplementary material for: Lifestyle can exert a significant impact on the development of metabolic comorbidities in early-stage colorectal cancer patients
Source: Front Nutr. 2025 Jul 4;12:1551526. doi: 10.3389/fnut.2025.1551526 (PMC12272228; doi:10.3389/fnut.2025.1551526)
Supplement: Supplementary file 3 [file Data_Sheet_3.docx]

Appendix B: Godin-Leisure Time Physical Activity Questionnaire

During one week, how many times on the average do you engage in the following kinds of physical activities for more than 15 minutes during your free time (Write the appropriate number in each line)?

|  | Times per week: |
| --- | --- |
| Strenuous Exercise (Heart Beats Rapidly)  Examples: Running, jogging, soccer, basketball, rope skipping, hiking, roller skating, strenuous swimming, strenuous long distance bicycling, cross country skiing, judo, hockey, squash. |  |
| Moderate Exercise (Not Exhausting)  Examples: Fast walking, popular and folk dancing, easy swimming, easy bicycling, table tennis, volleyball, badminton, tennis, aerobics, baseball, alpine skiing. |  |

Score = (9 × Strenuous Activities Times) + (5 × Moderate Activities Times)
